# Diagnosing myasthenia gravis versus congenital myasthenia gravis in British Shorthair cats: A case study

**DOI:** 10.14440/jbm.2024.0129

**Published:** 2025-04-21

**Authors:** Adriana Amfim, Maria Cartacuzencu

**Affiliations:** 1 Department of Veterinary Medicine, Faculty of Veterinary Medicine, Spiru Haret University, Bucharest 030045, Romania; 2 Veterinaty Clinic, Basarabia, Bucharest 030045, Romania

**Keywords:** Autoimmune, British shorthair, Congenital myasthenia gravis, *COLQ* gene, Diagnostic

## Abstract

**Background:**

Myasthenia gravis (MG) is an acquired autoimmune disorder characterized by autoantibodies targeting the neuromuscular junction of skeletal muscles. In contrast, congenital myasthenic syndromes (CMSs) represent a clinically diverse group of genetic disorders affecting the neuromuscular junctions, with early onset and autosomal recessive inheritance. CMSs are particularly prevalent in Devon Rex and Sphynx cats. The gold standard for diagnosing MG in cats involves detecting neuromuscular junction autoantibodies by measuring acetylcholine receptor autoantibodies using radioimmunoassay. For CMS, a definitive diagnosis requires the identification of a causative genetic mutation in addition to clinical signs of skeletal muscle weakness and fatigue. With the Sphynx and Devon Rex breeds, data collected have identified a candidate disease region on the feline C2 chromosome, discovered by employing a genome-wide single-nucleotide polymorphism-based homozygosity mapping strategy. Given that MG is an autoimmune condition, it is treated with steroids, immunosuppressive drugs, and sometimes a thymectomy (surgical removal of the thymus gland). CMS is a set of genetic conditions that do not respond to these treatments. Hence, accurate differential diagnosis is critical.

**Case presentation:**

Presented in this case study was a British Shorthair feline which was anamnetically, clinically, paraclinically, and pharmacologically assessed. Genetic testing revealed a positive result for the *COLQ* gene mutation.

**Conclusion:**

This case study clarified and added criteria to the differential diagnosis between MC and CMS, allowing for more accurate prognostic evaluations and appropriate treatment planning. It also underscores the importance of genetic testing in British Shorthair cats to differentiate between these conditions.

## 1. Introduction

Myasthenia, a syndrome of neuromuscular transmission, can present both as an acquired and congenital condition. The mechanism underlying myasthenia gravis (MG) is an acquired autoimmune disorder in which autoantibodies target the neuromuscular junction of skeletal muscles. Congenital myasthenic syndromes (CMSs) are a clinically diverse group of genetic disorders that affect the neuromuscular junctions and tend to have an early onset.^1^ These syndromes, which resemble but differ from MG, have been identified in dogs and cats since 1974.^2^,^3^ While autoantibody testing for acetylcholine receptors (AChRs) may yield negative results in affected animals (dogs and cats), they still exhibit symptoms such as skeletal muscle weakness and fatigue.

Congenital MG is characterized by activity-exacerbated neuromuscular weakness and is caused by a genetic mutation. It is primarily reported in cats of the closely related Devon Rex and Sphynx breeds. This pathology has not been confirmed in British Shorthair cats, or in other 14 breeds in a cohort study of 333 cats.^4^ CMS cats generally succumb to asphyxiation.

Congenital MG is an autosomal recessive neuromuscular disorder of neuromuscular transmission and was first described in the 1970s and 1980 in a Jack Russell Terrier breed and later in other dog breeds. This disease affects the presynaptic, synaptic, or postsynaptic regions of the neuromuscular junction. The pathogenic and genetic mechanisms of CMS involve mutations in the genes encoding the nicotinic AChRs epsilon subunit, choline acetyltransferase, and the collagen tail subunit of asymmetric acetylcholinesterase (COLQ). In cats, a form of CMS with a deficiency of *COLQ* has recently been reported in the Persian breed.^2^ CMS in cats is clinically characterized by fatigue and skeletal muscle weakness, and electromyographic or histopathological features of muscles can aid in diagnosis. Studies using a genome-wide single-nucleotide polymorphism-based homozygosity mapping strategy have identified a candidate region, *COLQ*, on the feline C2 chromosome. A homozygous c.1190G>A missense variant in exon 15 of *COLQ* leads to a C397Y substitution. Segregation of the c.1190G>A variant has shown 100% positivity, confirming an autosomal recessive mode of inheritance for the disorder. These results confirm that the neuromuscular disorder reported in the British Shorthair breed is a CMS caused by a single c.1190G>A missense mutation. Genetic testing now allows for the identification of these mutations, rendering early detection and prophylactic measures possible in this breed.^4^

Experimental autoimmune MG has provided a good model for studying the effects of the autoimmune response against AChRs in rodents and rabbits. Additional studies in dogs have shown that the most frequently monitored autoantibodies include those against AChR and a protein called muscle-specific kinase. In cats, autoantibodies have only been reported against AChRs.^1^,^3^,^5^

Future studies of autoimmune MG may help identify the factors that initiate and sustain the autoimmune response against AChRs.^6^ Before 2000, reports of feline MG were rare in the veterinary literature. However, in 1998, the Animal Medical Center of the Department of Medicine, New York, United States of America, conducted a study on four cats diagnosed with MG. Among them, one British Shorthair cat was found to have a congenital form of MG, while the other three cats – British Shorthair, Abyssinian, and Somali breeds – had the acquired autoimmune form.^7^ Although British Shorthair is one of the oldest breeds of cats in Great Britain, with Roman roots, the population significantly decreased after the Second World War. Mating programs with other breeds and inbreeding were extended until the 1970s. This led to the development of various phenotypic characteristics in the breed and the emergence of pathologies related to close inbreeding. Although these breeding programs have resulted in desirable physical and behavioral traits, they have also contributed to increased genetic defects and diseases.

Our study aimed to clinically evaluate and genetically confirm a presumptive diagnosis for some common and rare genetic diseases in British Shorthair as part of a more extensive screening epidemiological-genetic study. In the case of the British Shorthair cats from Romania, we considered rare genetic diseases, such as polycystic kidney disease, immune-mediated lymphocytic cholangitis, and meningomyelocele. There have been no recorded or published cases of congenital MG in this breed. For this reason, we established a 1-year genetic monitoring program for all the British Shorthair cats – with and without pedigree – in a small veterinary clinic in Bucharest to assess presumptive genetic pathology.

## 2. Case presentation

### 2.1. Case

This article presented a descriptive and observational clinical case evaluation focusing on a British Shorthair cat and incorporating anamnetic, clinical, paraclinical, pharmacological, and genetic approaches. This case was selected to establish specific differential diagnostic criteria that could be useful for practitioners. This is particularly relevant when standard methodologies, such as neuromuscular junction autoantibody testing, the gold standard for diagnosing MG, are unavailable, requiring genetic testing to confirm CMS. However, it is essential to note that these tests are not breed-specific, which can introduce limitations. In addition, we aimed to inform veterinary practice and highlight the potential challenges, limitations, and errors that clinicians may face when attempting to establish a definite genetic and differential diagnosis.

The study aimed to correlate commercial genetic test results approved for Sphynx and Devon Rex cats with clinical findings in British Shorthair. To address the common scenarios in veterinary practice, we specifically selected cats without pedigree, which adds complexity to the correct interpretation of a genetic test. We focused on the confirmation or no-confirmation for congenital MG, mainly because the commercial genetic test for *COLQ* gene runs is designed for Devon Rex and Sphynx breeds. Although this test can be applied to British Shorthair cats, it is not breed-specific, which raises concerns about the potential for false positive results in this breed.

For differential diagnosis in CMS and MG, only one cat, a spayed female British Shorthair, met the inclusion criteria out of the 10 non-pedigree cats screened over a period of 1 year.

### 2.2. Diagnostic methodology 1

The methodology combines several key elements of veterinary practice, including anamnetic considerations, clinical examination, paraclinical examination, radiological imaging, and additional pharmacological diagnostic techniques. The diagnostic methods are described below.

(i) Anamnetic considerations were performed using epidemiological approaches, such as species, age, sex, and examination period.
(ii) Clinical examination included temperature, a state of consciousness, coordination of movements, behaviors, and reflexes.
(iii) Paraclinical examination involved hematology, blood biochemistry, and ultrasound examination conducted in the clinic.
(iv) The veterinarian recommended a radiological examination (computed tomography) to rule out tumor formation (thymoma), but the owner refused.
(v) Additional pharmacological diagnostic was conducted to confirm the clinical diagnosis. The pharmacological test for MG was performed at the veterinary clinic with 0.02 mg/kg of Myostin (active substance: neostigmine methyl sulfate) administered intravenously. This product is known as Neostig in Germany, Prostigmine in Belgium, France, Luxemburg, Morocco, and Spain, and Prostigmina in Italy.^8^

In establishing a more precise clinical diagnosis, it would have been necessary to perform antibody testing and repetitive nerve stimulation. Unfortunately, neither method could be performed. These limitations, frequently found in the clinical field, hinder the certainty of a clinical differential diagnosis. Genetic testing would appear to be the most appropriate choice to certify genetic etiology, which provided that the test is approved for the specific breed or gene.

### 2.3. Diagnostic methodology 2

Genetic testing was performed to identify the causal mutation associated with CMS disease in the *COLQ* gene. The test is usually done for Devon Rex and Sphynx cats, but the laboratory can also conduct tests for other breeds upon request. For this test, 1 mL of peripheral venous blood was collected into ethylenediaminetetraacetic acid-coated vials, and the analysis was carried out by sequencing targeted known variants.

### 2.4. Outcomes

The cat presented with a neuromuscular transmission disorder with lethargy, inappetence, inability to swallow, hypersalivation, and loss of balance. On further conversation with the owner, it was found that the cat had previously visited another veterinary clinic, where a potential flower toxin exposure was suspected. To specify the clinical diagnosis, a thorough differential diagnosis was necessary, which included consideration of conditions such as myopathy, neuropathy, MG, congenital MG, *Toxoplasma gondii* infection, hyperthyroidism, cranial tumor formation, and vestibular syndrome.

Anamnetic considerations showed that the cat was from the feline species and was a British Shorthair breed without a pedigree test. The cat was a 1.2-year-old spayed female weighing 3.9 kg. The examination was conducted on March 10, 2023. The clinical examination revealed that the cat’s body temperature was 37.4°C, with pale mucous membranes and a heart rate ranging from 168 to 185 beats/min. The capillary refill rate was two seconds, and the respiratory rate was 25 breaths/min. The lymph nodes were reactive and explorable, and muscle mass was assessed as reduced, with a low level of dehydration noted. The cat also displayed a depressed attitude. A consultation of the central and peripheral nervous system was carried out, revealing the following:

(i) State of consciousness: Present
(ii) Coordination of movements: Cranial ventroflexion, ataxia, inability to maintain a quadrupedal position, limb extension, and absence of proprioception.
(iii) Behaviors: Lethargy, inability to support weight on limbs, generalized muscle weakness, and vocalization when handled.
(iv) Reflexes: Abolished pupillary, palpebral, defense, normal right eye reflex, and gluteal reflex present.

The biochemical results, showing elevated levels of specific parameters, were obtained following the evaluation of the biochemical parameters on March 10, 2023. The other remained within the physiological range. The elevated biochemical levels are presented in Table 1.

**Table 1:** Evaluation of biochemical parameters

Parameter (physiological values)	Results
Albumin (1.9 – 3.5 g/L)	3.8 (elevated)
Amylase (370 – 2,229 U/L)	2,373 (elevated)

A pharmacological diagnosis for MG was performed at the veterinary clinic to clarify the diagnosis. Myostin, at 0.02 mg/kg, was injected intravenously, with positive response observed after administration. There were head movements, and the cat could maintain a sternal position without ataxia. The reflex returned, and she could swallow with appetite present, with no regurgitation, and maintain a quadrupedal position with heavy walking. The active substance for Myostin is neostigmine, a substance analogous to pyridostigmine.

We proceeded with a pharmacological test in a case with a presumptive MG diagnostic for a rapid differential diagnosis according to the present symptomatology. After administration of the anticholinesterase agent (Myostin), the response was positive, and the cat’s condition improved visibly following the treatment at the office and home. Regurgitation episodes were rare; she started eating independently and showed no recurrence of muscle weakness. However, on March 1, 2023, the cat died suddenly of asphyxiation due to choking on food. The clinical picture, the chronic and progressive nature of the disease, and the cat’s response to the anticholinesterase drug could confirm the presumptive diagnosis of MG.

**Figure 1:**
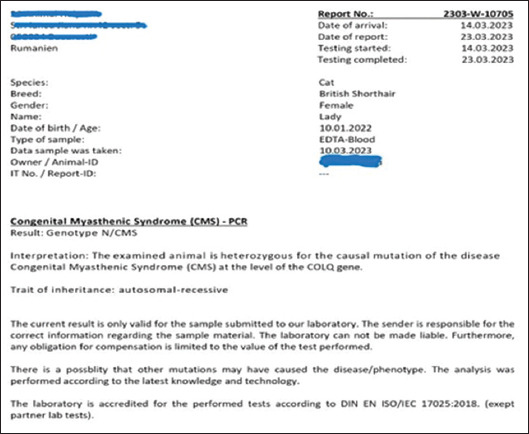
Result of genetic testing for congenital myasthenic syndrome. The genotype N/CMS indicates that the cat was heterozygous for the condition.

A genetic examination was conducted using a COLQ single gene test, which involved sequencing known variants targeted according to the specifications provided by the commercial laboratory. The genetic test result confirmed a diagnosis of CMS, with the genotype N/CMS, indicating the cat was heterozygous for the causal mutation of CMS in the *COLQ* gene. The inheritance pattern was identified as autosomal recessive (Figure 1).

The association between the *COLQ* variant and CMS allows clinicians to confirm diagnosis through genetic testing and permits owners and breeders to identify carriers in the population. Moreover, accurate diagnosis increases the available therapeutic options for affected cats based on a better understanding of the pathophysiology and insights from human CMS cases associated with *COLQ* variants.^9^ However, veterinary literature remains limited on this topic, and while the etiopathogenesis of CMS remains speculative, an immune-mediated pathogenesis is suspected. The genetic background, as seen in this condition, is shown in Table 2.

**Table 2:** Characteristics of myasthenia gravis and congenital myasthenic syndrome in cats

Disease	Myasthenia gravis	Congenital myasthenic syndrome (CMS) in Devon Rex and Sphinx
Mendelian trait/disorder	Unknown	Yes
Considered a defect	Yes	Yes
Gene symbol	Not available	*COLQ*
Description	Autoimmune response against acetylcholine receptors at the neuromuscular junction	Genotype N/CMS is a carrier, and genotype CMS/CMS is affected
Inheritance pattern	Unknown	Autosomal recessive
Other names	Myasthenia in *Felis catus*	Muscular dystrophy-dystroglycanopathy (limb-girdle) in *Felis catus*

## 3. Discussion

In our observational case study, the condition in the cat presented at an early age of 8 months, beginning with an inability to swallow and hypersalivation, followed by loss of balance and mild generalized skeletal muscle weakness, especially after exertion, stress, or excitement. The generalization, progression, and severity of skeletal muscle weakness and fatigue reflect the clinically heterogeneous congenital syndromes affecting the neuromuscular junction. By the time, the cat was presented to the veterinary clinic at 1 year and 2 months of age, the weakness of the skeletal muscles was generalized and severe, with the palpebral reflexes absent.

In the context of a presumptive CMS diagnosis, it is essential to confirm the condition with a genetic test. A heterozygous result (N/CMS) in Devon Rex and Sphynx represents an asymptomatic carrier state, while the homozygous (CMS/CMS) state leads to the disease form. In our study, the cat was heterozygous (N/CMS) for the disease-causing mutation in the *COLQ* gene. Although the genetic test suggested an asymptomatic carrier state, the cat exhibited symptoms, raising the possibility that an unidentified genetic abnormality may play a role in the development of congenital MG in this British Shorthair cat. While autoantibodies against AChRs are involved in the autoimmune response and pathogenesis of congenital MG, the deficiency in the *COLQ* gene also contributes to the disorder. Due to the chronic nature of the pathology and the progressive muscular repercussions, the response to treatment may be delayed or absent, which was the case with this cat, who tragically succumbed to sudden death.

Studies on MG in cats remain scanty, primarily due to a high euthanasia rate for cats with the condition. Research suggests that congenital MG or CMS is subject to both environmental and genetic factors, with Devon Rex and Sphynx breeds being more prone to the condition. These breeds often exhibit common inbreeding practices, though no sexual predisposition has been identified. Despite significant progress in the understanding of acquired MG in companion animals over the past decade, the mortality rate in dogs and cats with acquired MG remains unacceptably high.^10^ Identifying a causative genetic mutation and clinical signs is necessary to establish a definite diagnosis.^1^ The heterozygous result of the genetic test in this British Shorthair cat could represent a novel finding for the breed, as only homozygous forms of the mutation have been shown to manifest CMS symptoms in other breeds such as Devon Rex and Sphynx.

## 4. Conclusion

In this study, we have established three significant limitations in the differential diagnostic process for this case. The first limitation was the owner’s refusal to subject the cat to radiological examination (computed tomography). If the radiological examination had been performed, the tumor could have been identified, supporting the diagnosis for MG instead of CMS. The second limitation was the lack of AChR autoantibody concentration testing using radioimmunoassay, the gold standard for diagnosing MG in cats. Unfortunately, both these situations are common in clinical practice and must be acknowledged as significant factors in diagnostic decision-making. The third limitation concerns using a genetic test recommended for other breeds, such as Devon Rex and Sphynx. In our case, using this test for British Shorthair cats led to a potential for false results. This highlights the importance of exercising caution in the interpretation of the genetic test results, especially when breed specificity is not established, as with the *COLQ* gene mutation. Furthermore, without a genetic test for pedigree confirmation, it was unclear whether the cat was truly a pure British Shorthair, even though its phenotype resembled the breed’s. This potential gap in the genetic interpretation adds more uncertainty to the diagnosis.

Given these limitations, we believe that practitioners must be aware of these factors when establishing a differential diagnosis. The absence of imaging examination that could have revealed a thymoma (for MG) and the result of the genetic test (heterozygous) for the *COLQ* gene mutation hindered the ability to draw definite conclusions about the severity of the disease or the exact form it took. However, age is an essential criterion for diagnosis. CMS typically begins at around 3 weeks of age. In contrast, this cat’s symptoms started at 8 months of age, supporting the presumptive diagnosis of MG rather than CMS, despite a positive genetic test for the *COLQ* gene.

Given these considerations, we advise clinicians to be cautious when choosing diagnostic methods and to have thorough discussions with the owners. We strongly recommend the gold standard method be used for MG diagnosis, especially in the absence of a genetic test approved for the breed and specific pathology like CMS.

In addition, due to the limitations of this single case study, no definite conclusion can be reached about the genetic basis of this disease in the British Shorthair breed. Further investigation with a significantly larger sample size and comparative studies between purebred British Shorthair cats and breeds such as Devon Rex or Sphynx cats is required before any definitive conclusions regarding the genetic predisposition to this pathology can be drawn.

## Data Availability

The epidemiological dataset was based on the owner’s report, collected through a epidemiological data under the protection of the General Data Protection Regulation. Data of this study are available from the corresponding author on reasonable request.
